# Developing oral health services for people experiencing severe and multiple disadvantage: a case study from Southwest England

**DOI:** 10.3389/froh.2024.1283861

**Published:** 2024-04-24

**Authors:** Martha Paisi, Lyndsey Withers, Rebecca Anderson, Janine Doughty, Lisa Griffiths, Ben Jameson, Elizabeth Murphy, Afsha Musa, Abigail Nelder, Shona Rogers, Robert Witton

**Affiliations:** ^1^ Peninsula Dental Social Enterprise, Peninsula Dental School, University of Plymouth, Plymouth, United Kingdom; ^2^ Community Research Partner, Plymouth, United Kingdom; ^3^ School of Dentistry, University of Liverpool, Liverpool, United Kingdom; ^4^ Health Inclusion Pathway, Plymouth, United Kingdom; ^5^ Improving Lives, Plymouth, United Kingdom

**Keywords:** health inequalities, dental health services, homeless persons, participatory research, oral health

## Abstract

People experiencing severe and multiple disadvantage (SMD) have disproportionately high levels of dental disease and tooth loss but have limited access to dental care. This paper presents an evidence-based case study of co-designing, implementing, evaluating and refining a community dental clinic for people experiencing SMD in the Southwest of England. It shares challenges, lessons, and solutions. Tailored interventions that coordinate flexible and responsive care are important for facilitating dental access for individuals experiencing SMD. Participatory approaches can deliver a range of impacts both on research and service development. No single fixed model of co-design can be applied in service development, and the choice will vary depending on local context, available resources and joint decision making. Through co-design, vulnerable populations such as those with SMD can shape dental services that are more acceptable, appropriate and responsive to their needs. This approach can also ensure long-term sustainability by bridging treatment pathway development and commissioning.

## Introduction

### Nature of the problem calling for innovation

Homelessness, problematic substance use, and repeat offending overlap considerably and are key characteristics of severe and multiple disadvantage (SMD) (1, 2). People experiencing SMD are likely to suffer significant health problems, and be heavy users of emergency services (3–6). They exhibit frailty and die some 30 years earlier than the general population, yet encounter personal and institutional barriers to using health, social and housing services (3, 6–8).

**Table 1:** SWOT analysis of the service.

Strengths	Opportunities
- Dedicated co-ordinator processing referrals, booking appointments etc. - Support staff engaged with the programme e.g., accompany patients - Links with support organisations - Outreach visits to break down barriers - Flexible attendance policy - Embedded evaluation - Integration with other health services	- Continuous learning through service evaluation - Introduction of a patient passport - Trauma informed practice and organisational change - Dental workforce education - Stimulation of broader health engagement by patients - Cross referrals to other services - Education and outreach opportunities for students
Weaknesses	Threats
- Missed appointments and lost clinical time - SMD patients’ reliance on emergency care - SMD patients’ low readiness to engage with routine dental services.	- High demand and nature of SMD - Overwhelming number of referrals - Limited clinical time - Difficulties in contacting patients - Patient anxiety - Strain on wider dental care system

The most socially excluded people experience a “cliff-edge” of stark and persistent inequality, not least in their experience of oral disease (9). They are disproportionately affected by rampant caries, periodontal disease and tooth loss, as well as increased risk of oral cancer (10–13). Their complications of dental disease commonly include dental or orofacial pain, abscesses and infections (14). Oral disease is one of the top five reasons for hospitalisation among people who use heroin (15).

Despite their greater needs, people experiencing SMD are not able to access universal services in an equitable way with a proportionate response from health and social care services (16, 17). Even when the intent is to deliver a responsive service, capacity issues can disadvantage those most in need via the “inverse care law” (18, 19).

Barriers to accessing and receiving timely dental care stem from both the lived experience of SMD and the healthcare system, commonly resulting in late presentation of disease and visits to Emergency Departments for otherwise preventable conditions (16, 20). This has far-reaching impacts on physical and mental health, food intake, and ability to function in everyday life (21). It leads to low self-esteem, stigma, social isolation, and reduced employability (10, 11, 22). As a result, patients may also use drugs and alcohol to cope with dental pain, leading to further deterioration of dental health and perpetuating the SMD cycle (2, 21, 23).

Because oral health problems occupy a crucial position in the life of those experiencing SMD, dental treatment can catalyse benefits in multiple areas of a patient's life (24, 25). Contact with dental health services can offer an entry point to engage with other health and support services such as drug and alcohol rehabilitation, smoking cessation, and immunisations. Dental care can boost morale and self-esteem, opening up pathways to improved overall health, as well as training and employment opportunities (24, 25).

### Context in which the innovation occurs

Community-based participatory research is a form of co-design that unites science and practice through community engagement and social action to deliver increased health equity (26). Participatory research is a philosophy in which the research is done *with* those who are its focus rather than done *on* or *to* them (27–29). It is a paradigm, not a method, that guides the research process, emphasising power sharing, which is particularly relevant to socially excluded groups (27, 30). In their framework to promote oral health inclusion (31), Freeman and colleagues called for an evidence-based action plan informed by mixed research methodologies and underwritten by participatory research concepts. Co-design gives privileged exposure to the voices and lived experience of people experiencing social exclusion and consequent health disadvantage (31).

Both professionals and service users are directly affected by the quality of services offered by a healthcare system, and both need to be engaged in related research (27). The views of people affected by SMD on optimal outcomes of dental care or service use may differ from those of providers (32). Therefore, involving them in research promotes contextually sensitive interventions and appropriate approaches to patient care (33). Yet, oral health service design and policy targeting people experiencing SMD have only limited insight from the lived experience perspective.

Considering the burden of oral disease among people experiencing SMD and the disparity between service need and utilisation, facilitating timely high-quality care for them is essential. This is in line with the Long-Term Plan of the UK National Health Service (NHS) (34), which places priority on the health care of individuals with additional needs. The importance of integrating health and social care services for people experiencing SMD is recognised in strategic statements and guidelines (17). In addition, new planning structures in the NHS in England, called Integrated Care Boards, may offer fresh opportunities to commission place-based health inclusion models that design care around the needs of specific groups, and help spread innovation and best practice (35).

## Purpose

This paper presents an evidence-based case study involving co-designing, implementing, evaluating and refining a dental service for people experiencing SMD in the Southwest of England, sharing key lessons from a partnership of stakeholders.

## Methodological approach

This is a community case study which documents local experience in developing a dental service for people experiencing SMD. It describes and reflects upon, a programme and practice geared towards improving the health and functioning of this cohort. The Community Dental Clinic was established in early 2018 by the Peninsula Dental Social Enterprise (PDSE), the clinical arm of the Peninsula Dental School at the University of Plymouth, Southwest England. PDSE aims to improve oral health and reduce inequalities by provision of quality care to groups who find access to mainstream services challenging (36).

PDSE identified the need to improve access to dental services for one such group, i.e., people experiencing homelessness. It shaped its response by developing care pathways suited to their needs and circumstances based on a range of inputs from diverse data sources. These included community engagement activities, on-the-ground experiences, consultation with local stakeholders, evidence synthesis and primary studies to assess the oral health needs of the local population experiencing SMD and their barriers to care. Stakeholders included dental and other healthcare professionals, university and peer researchers, community representatives, patients, and support workers. Participatory research values guided the process throughout, giving all contributors the opportunity to input ideas. Thereafter, through an iterative process, all authors contributed to and refined the emerging themes to present in this case study.

### The history of the innovation

A systematic review of barriers and facilitators to accessing dental care for people experiencing homelessness in the UK found linkages to both the lived experience of homelessness and the nature of the healthcare system (16). The review recommended reconfiguring future services to recognise the target group's diverse and complex needs. Building on these findings, the PDSE academic team and peer researchers from the charity Groundswell collaborated in 2018 in a qualitative study at a homeless hostel (37–39). Groundswell works with people experiencing homelessness and other disadvantages, enabling them to participate in decision making and help create solutions in areas including health (40). The partners paid an informal familiarisation visit to the hostel prior to the data collection and intervention, giving an opportunity to meet residents and share views on oral health.

The study investigated factors influencing oral health behaviours and access to dental care from the perspective of people with lived experience of homelessness plus stakeholders including support workers, dental providers and other health professionals. The results were used to develop an oral health intervention project and feed into the development of the PDSE Community Dental Clinic.

Peer advocates were involved at every stage from the study design to the planning and delivery of the oral health promotion intervention, including data collection from people experiencing homelessness, and evaluation, interpretation and dissemination of findings. Other stakeholders (hostel support staff and other professionals in various supporting roles) were interviewed by a member of the academic team. Data collection focused on discovering what was considered important regarding oral health promotion and optimal dental service provision.

### Realising a co-designed dental service

In response to our study findings and in line with Freeman and colleagues' inclusion oral health framework (31), PDSE established a dedicated dental pathway for people experiencing homelessness to fill identified gaps in service provision (24). At its launch, the PDSE Community Dental Clinic was a pro-bono contribution to the local community (24). A salaried dentist provided routine and urgent treatment, all without cost to patients. Subject to patient consent, appointments were arranged in coordination with support staff or volunteers who provided appointment reminders, transport to the clinic, and chaperoning during treatment, as needed.

Recognising the voluntary sector as an important partner for statutory health services, supporting improved health, well-being and care outcomes (41), a close collaboration was established with a local volunteer with years of experience in the homelessness sector. This helped ensure that voices from that sector were continuously heard in developing and delivering the service.

### Evaluation and iterative redevelopment of the service

Following establishment of the Clinic's initial model of care, an outcome and process evaluation was carried out in 2020 to determine its impact and acceptability for patients, and examine barriers and facilitators to using and providing the service (24). Based on the evaluation findings, plus community outreach experience and opportunities created by the local commissioning organisation (NHS Devon) funding the service (42), some changes have been put in place since October 2022. These address identified gaps and recommendations for improvement:

1) Referral criteria: These have broadened from those experiencing homelessness to those experiencing SMD. Referrals are now accepted from any of the seven organisations in the Plymouth Complex Needs Alliance (43).
2) Patient documentation: A bespoke patient information sheet and referral form have been developed to enable tailored care.
3) Referral and appointment process: The PDSE Dental Outreach Team (as opposed to PDSE Administration) receives referrals via a dedicated email address, processes them, and communicates appointments.
4) Outreach activity: The Dental Outreach Team visits referring organisations to meet prospective patients in an environment where they feel comfortable to introduce dentistry in a positive way, and reduce stigma and apprehension.
5) Integration: Active integration has been established with supported housing, and social and health services.
6) Patient satisfaction: A satisfaction questionnaire is administered to patients completing treatment.
7) Patient and Public Involvement (PPI): A PPI group comprising people experiencing SMD, support staff and other professionals has been established to improve service delivery and identify further opportunities to support the community. They will contribute to a mixed methods evaluation of the service to explore factors influencing integration with other health and social care services.

### The patient journey

**Figure 1:**
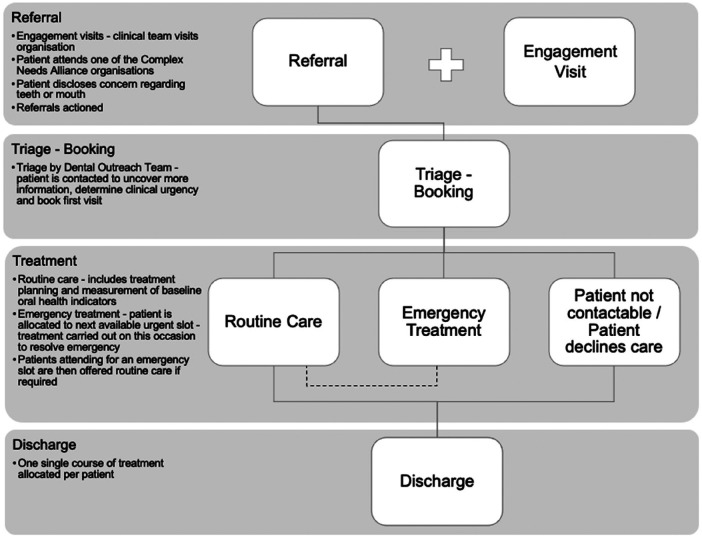
Patient journey.

Once a referral has been received at the dedicated email address, the patient is registered, and patients and/or support staff are contacted (depending on consent) to ascertain clinical urgency and communicate appointments (see Figure 1).

Two days prior to the appointment date, a text reminder is sent to the patient and copied to the respective community organisation. Support staff are encouraged to accompany patients as needed.

Each patient is offered an assessment and one complete course of treatment by a fully qualified dental clinician. Those requiring urgent treatment are allocated an emergency appointment to resolve the immediate concern before moving to routine treatment.

Patients who cannot be contacted via their support worker or community organisation, or who fail to attend two visits, are discharged from the service, but can be re-referred once they are in a position to undertake treatment.

To avoid the disappointment and de-motivation of a long lead-in time, once clinic capacity is reached, the waiting list is shut. Support organisations are informed of the opportunity to make referrals again when capacity becomes available. Notwithstanding this, two emergency slots for patients experiencing SMD and requiring urgent treatment remain available each week.

## Lessons learned—recommendations

During the co-design, delivery, evaluation and redevelopment of the above service, we have learned many valuable lessons that we believe will support others who wish to create inclusive dental services for SMD groups. Below are key lessons that have been identified through a group discussion and refined over time, using various data sources described earlier (i.e., in “Methodological approach”). Both the SWOT analysis (Table 1) and other key lessons learnt reflect facilitator-led group exercises.

The concepts distilled into the SWOT analysis are developed below.

### Effective partnerships

In our work, all collaborators in the participatory research partnership hold equal positions in the team. Prerequisites for effective collaboration include collectively setting clear goals and expectations, power sharing, encouraging joint working, and valuing individual contributions and differences (44). These are fostered by establishing transparency, creating a friendly setting and conditions for trust, and building relationships and agency for all, with training where appropriate.

The diversity of people experiencing SMD should be reflected in the co-researchers. Their individual skills and capabilities may vary, calling for support from academic colleagues to help them fulfil their roles (44). Co-researchers with lived experience are likely to have been exposed to trauma in similar circumstances to the research participants. It is important to be mindful of their wellbeing, with appropriate support mechanisms and regular debriefing (45).

### Tailored innovations facilitate access

Provision of general dental care in England has experienced progressive strain, resulting in significant access constraints (46). This is particularly acute in Southwest England. Moreover, service commissioning and delivery models are designed for the general population, lacking the flexibility to accommodate complex lives and needs (47). Previous incentives from NHS comissioners have not always had successful uptake, possibly for not accommodating challenges in reaching Units of Dental Activity targets, which are a common feature of dental contracts in England.

Access to care for people experiencing SMD should meet immediate needs alongside building personal resources and resilience to achieve a happy and healthy future (i.e., “recovery”). Teeth often tell the tale of a life filled with attrition, and only through recognising the importance of planned and supported dental care alongside mental and physical health care can recovery be fully supported.

The current “one size fits all” model of dental access does not consider the underlying factors perpetuating the oral health equity gap for this cohort, carrying the risk that any interventions developed will fail those who are the most vulnerable (14, 18). A dedicated clinic that operates with the flexibility required to meet complex needs and lives can mitigate that risk. Box 1 lists some characteristics of an environment within which the service can flourish, as suggested by patients and other stakeholders through research and practical experience.

BOX 1 **Developing an oral health service for SMD patients.**

An oral health service for people experiencing SMD should meet three requirements: (i) prevention and access to dental hygiene, (ii) a responsive service for emergencies, and (iii) high-quality restorative care to support recovery. This would include

• Situation-appropriate support for self-care:
  • access to clean running water
  • provision of sanitary spaces
  • access to dental supplies in hostels and drop-in centres
  • education about dental hygiene
  • reinforcement of good routines.
• Linkage to dental services taking into account patients' circumstances:
  • dental treatment offer prioritised and matched with need and readiness for treatment
  • a timely response through sufficient capacity to offer emergency and ongoing treatment
  • ability to locate and communicate with people who may not have a fixed abode or access to a mobile phone
  • accessibility through multiple services within which professionals are aware of a simple referral process
  • peer support and flexible support in the community to foster and build engagement.

### Coordinating flexible and responsive care

Most operational challenges to running the PDSE Community Dental Clinic stem from the high demand and the nature of SMD. The number of referrals received can be overwhelming, often exceeding available clinical time and requiring prioritisation of patients in discussion with the clinical team. Reaching patients, particularly those with no fixed abode and possibly without a mobile phone or credit, can be difficult, leading to reliance on support organisations/workers to make contact. Patients with co-morbidities and poor mental health often find attendance challenging. Some are very anxious and need help to ensure and sustain their attendance.

Having a dedicated co-ordinator processing referrals, booking appointments and responding to emergencies and cancellations helps establish a relationship with patients and support staff, facilitates communication with patients, and improves service efficiency. Outreach visits to meet potential patients in their own environment promote good working relationships with support organisations and patients, and break down barriers. Adaptability and empathy in the face of unforeseen situations, and flexibility around appointment timing may be crucial for patients with addictions who follow certain medication routines and/or who may risk withdrawal unless accommodated. Longer appointments support building of trust and confidence for anxious patients, explanation of procedures, and agreement on treatment plans.

Further suggestions from patients and support workers are given in Box 2 below.

BOX 2 **Patient and support worker feedback on the clinic environment**

• Have clear signage pointing to and on entrance doors to help new patients.
• Provide low music to soften “the overbearing silence” in the waiting room.
• Have a TV with subtitles in the waiting room to help distract and calm nerves.
• Display photographs of the dentists without masks “to know what their smiling faces look like”.
• Put activity-focused pictures on ceilings/walls in clinical spaces to divert the mind away from the treatment.

### Supporting patients to utilise services

A key factor for an effective service is minimising clinical time lost through missed appointments. Whilst failure to attend is to some degree inevitable across all patient groups, those experiencing SMD often have chaotic lifestyles, making communication around appointments more difficult and missed appointments more likely (16). Discussing and recording consent for sharing information with support services from the outset can allow service providers to liaise with them to remind patients and reschedule appointments if the patient is not able to attend.

A baseline policy on patient attendance, applied with a degree of flexibility, can help establish expectations from the beginning and ensure consistency. Discussing the reasons for a missed appointment with the patient or their support worker can help accommodate lifestyle factors in treatment planning and increase the chances of success. Knowing where a patient is in their addiction and recovery journey may have implications for their ability to embark on extensive treatment plans requiring multiple appointments, as opposed to only receiving urgent care.

Introducing a “Patient Passport” accessible to service providers could assist SMD patients in recalling pertinent health information, alleviate pressures on them to retell traumatic experiences, and allow them to flag personal likes and dislikes about health treatments.

### Support staff role

Through spending a lot of time supporting people with health and social care needs, homelessness support workers hone their skills in handling difficult conversations, recognising important conversational cues, and building relationships with clients who distrust other professionals (47, 48). However, since people experiencing SMD rely heavily on emergency care, workers may tend to focus less on initiating general and prevention-oriented health conversations (49). Research is needed to investigate how to enhance their confidence, skills and knowledge to have more effective conversations, achieving improved signposting and healthcare advocacy (48). Encouraging people to start speaking about health issues, including oral health, can start a journey to a healthier life based on higher health aspirations, self-advocacy and ability to support themselves in the future (48).

### Outreach improves patient engagement

Outreach visits provide the dental team with a greater depth of knowledge and understanding of the day-to-day challenges that patients and support workers encounter. Hence, we organise frequent visits to the community. People experiencing SMD are no strangers to shame, oral health-related stigma and dental anxiety (16, 50). So, reaching out to them at places where they feel safe and comfortable (e.g., residential programmes; drop-in centres) and through street outreach helps break down barriers of fear and anxiety (38, 39). Triaging on outreach visits achieves introduction to the clinical environment and personnel in a relatively gentle way.

### Trauma-informed practice is a priority

People with complex lives and needs related to homelessness and other aspects of SMD are highly likely to have experienced trauma and stigmatisation in both healthcare encounters and interactions with society at large (16, 21, 24, 51). Adverse past experiences with health services can have a profound impact on patients’ engagement (13), sometimes manifesting as behaviour outside what would otherwise be considered acceptable in a clinical setting. However, negative reactions from staff to challenging behaviour can deter patients from attending future appointments.

There is evidence that engagement is promoted through approaches that are friendly, non-judgemental, and culturally sensitive (17). Our ongoing evaluation of the Community Dental Clinic and feedback demonstrate the importance of patients being treated with respect and humanity, first and foremost as a person to be helped rather than as a problem to be solved. Practical measures including sensitive waiting room arrangements providing adequate privacy, information management to avoid patients having to repeat personal details which may be retraumatising, tactful offers of assistance with filling in forms, and space on referral forms to alert the dentist to any additional needs or experience of past trauma requiring an appropriate and sensitive response.

Trauma informed approaches are increasingly recommended as a means to empower individuals to participate in their own healthcare and thereby promote better outcomes (34, 52, 53). However, an evidence base for the effectiveness of such approaches in the dental sector is limited, justifying further research.

### Dental workforce—education

Dental professionals require a holistic and empathetic understanding of the entire patient population, and dental faculties must realise their responsibility to orient educational and research activities to society's current and future health needs (54). Education systems train graduates to be competent in diagnosis, treatment planning and technical skills. However, the limited integration of outreach into traditional dental curricula means that the mind-set of graduates is not often community oriented. Dental educators should promote an understanding of inclusion health, with practical opportunities for students to work with marginalised populations.

The Inter-Professional Engagement programme at the Peninsula Dental School provides such an approach (55). Through experiential and peer learning, the students develop insight into community-wide patient care needs. The students graduate with a truly rounded set of skills, taking awareness and openness to innovation into their professional careers.

### Embedded evaluation—being a learning institution

Services benefit from embedding evaluation into their workplans to create learning opportunities from the outset. Documentation of the use of the PDSE Community Dental Clinic and engagement by partner organisations and patients has provided a clear picture of the dynamics of the patient population, their use of the service, and its acceptability. It has identified gaps and opportunities in service provision, leading to changes in operation to better serve patient needs. Further research on identifying patient-centred data and indicators of “small” or “soft” outcomes that are meaningful to individuals will enhance understanding of patient experiences (56).

### Integration with wider healthcare and SMD services

With high levels of multimorbidity and social care needs among people experiencing SMD, interdisciplinary working is an effective and productive way of organising care around the individual, drawing on greater awareness of the interaction of homelessness and health, and cross-referrals among services. This is consistent with the joint guideline published by the National Institute for Health and Care Excellence and the Centre for Homelessness Impact (17).

The PDSE Community Dental Clinic currently operates in partnership with the Health Inclusion Pathway, Plymouth. This model of multidisciplinary service provision coordinates care across outreach, primary, secondary and emergency healthcare, social care and housing services for people experiencing SMD. By embedding access to dental care in a service directly aimed at this population group, it is hoped to make it more easily available and more readily engaged with.

## Discussion

We have explained how we have used co-design to develop an effective, responsive dental service for SMD groups, and have reflected on the lessons learned through the design, delivery and evaluation of the service. The acceptability and appropriateness of the service were evidenced by all stakeholders (patients, providers, support staff, researchers) through formal and informal feedback (e.g., evaluation of the service, feedback questionnaires, stakeholder group meetings and PPI) and rate of attendance at the PDSE community clinic. Acceptability and appropriateness are interrelated, and include considerations about the opportunity for individuals to participate in their own care and be empowered to make decisions (57). This includes meeting their cultural values and norms while addressing their health needs (58).

### Reflections on participatory research

A number of studies throw light on approaches to co-design (33, 59, 60). Our approach is similar to “experience-based co-design”, which collects user experiences and uses them to formulate interventions or pathways (61). In this approach, stakeholders are recognised as possessing both explicit and tacit knowledge; working together in a group helps surface the latter and facilitates the creation of new shared meaning visible to all stakeholders (33, 60). In our work, people with lived experience were involved separately from other stakeholders (e.g., support staff, clinicians) to minimise discomfort caused by power dynamics. To look at this further, through PPI, we are exploring the possibility of incorporating “experience-based co-design” more fully into service development.

### The role of participatory research

People experiencing SMD often have low health expectations and commonly have decisions made on their behalf, stifling their opportunity to exercise agency (62). Yet from our experience, the extensive knowledge that people with lived experience have of the structures behind SMD equips them to be a vital part of the solution, contributing at practical, policy and political levels. Involving them can help ensure the acceptability, appropriateness, effectiveness and sustainability of services. It can help build patients' trust in services and service providers (17) and enable them to become partners in their dental treatment rather than simply recipients of care.

Also important are the views of other stakeholders, making the research a partnership involving academics, service users, and dental care professionals, as well as workers from the third sector and other health and social care fields (27).

### Benefits of participatory research

The value of participatory research cannot be overstated. There is no current standardised description of its impact, but studies have identified common effects including improved research plans, learning among partners and academic researchers, and impact on policy makers (63). Increased relevance of research to patients, healthcare professionals and other end users is undeniable, avoiding research waste (64).

Formal and informal feedback processes documented benefits felt by all of our stakeholders through improved study quality, relevance of research tools and outcomes to end-users, and generation of more honest research data. Deeper insight was gained into the factors that affect access to dental services by those experiencing SMD, because patients reported feeling more comfortable speaking to peer researchers who “had been where (they) are”, and thereby better able understand them (37). The academic team gained wider perspectives and knowledge of the subject area and of participatory research itself. In line with findings from other studies (44), co-researchers benefited through acquiring new skills, personal development and improved confidence. Individuals experiencing SMD gained improved access to treatment.

### Challenges and drawbacks of participatory research

The lack of methodological and terminological standardisation surrounding participatory research, and the lack of practical knowledge on how it can best be designed and performed, poses challenges in conducting meaningful public and stakeholder engagement (65). A better picture is needed of how it can best be performed in the context of dental service development. As suggested by the International Collaboration for Participatory Health Research (27), the appropriateness of any given model of participation is dependent on local context, available resources, and joint decision making. A lack of clear conceptualisation could be addressed by sharing lessons and experiences of what actually happens, reflecting on the process of participation and collaboration, and capturing positive outcomes, challenges and negative experiences (65, 66).

In line with published research (44, 64), we found that participatory research required increased time inputs and costs, and substantial flexibility and effort by all team members to foster partnerships and motivation. Negotiating power dynamics and/or relinquishing power can be challenging, along with balancing differences in perceptions, priorities and preferences, which may result in compromised study designs and apparent tokenism (67). Forming partnerships at an early stage can help with power relationships, with active listening on the part of the academic team to make partners feel heard, and assigning clear roles from the outset to help mitigate tensions. There is also a need to build trust through consistency and explicitly actioning recommendations made by those with lived experience.

Clinical governance regulations may impede adherence to principles of participation in health services evaluation, calling for organisational changes to improve capacity for partnership work. For example, interviewing patients without being employed by dental services may be challenging because of issues of confidentiality.

## Conclusions

Current dental policy in England fails to address the needs of people experiencing SMD. As a result, oral health inequalities continue to widen. Developing and providing more equitable and inclusive dental care pathways will necessitate much greater recognition of the needs of this cohort, including multi-disciplinary input and additional service requirements. Alongside this, more work is required on developing appropriate funding models so that dental providers and teams have the flexibility and capability to provide urgent and routine care in a sustainable way.

Our case study confirms co-design as a powerful approach with the potential to provide socially excluded populations with services that are more appropriate, acceptable, and responsive to their needs, at the same time as meeting providers' capabilities. Moreover, it allows for a bridge between treatment pathway development and commissioning, ensuring long-term sustainability of services.

Our long-term vision is for a radical system change that would recognise the need to prioritise vulnerable groups in the community. There is a need to explore a radical service redesign involving people with lived experience, incorporating research on how co-design can best be utilised. Services are likely to fail if they are simply transactional, using people as passive units of service need rather than being redesigned with people and taking into account how they live their lives. Their voice is important in co-designing and co-producing services that will offer excellence in treatment, provide equitable and tailored access, and achieve optimal outcomes in line with specific needs.

## Data Availability

The original contributions presented in the study are included in the article/Supplementary Material. Further inquiries can be directed to the corresponding author.
